# Gluten consumption and inflammation affect the development of celiac disease in at-risk children

**DOI:** 10.1038/s41598-022-09232-7

**Published:** 2022-03-30

**Authors:** Renata Auricchio, Ilaria Calabrese, Martina Galatola, Donatella Cielo, Fortunata Carbone, Marianna Mancuso, Giuseppe Matarese, Riccardo Troncone, Salvatore Auricchio, Luigi Greco

**Affiliations:** 1 Department of Translational Medical Science, University Federico II, Via S. Pansini 5, 80131 Naples, Italy; 2 European Laboratory for Food Induced Diseases, University Federico II, Via S. Pansini 5, 80131 Naples, Italy; 3 Department of Clinical Medicine and Surgery, University Federico II, Naples, Italy; 4 Laboratory of Immunology, Institute for Experimental Endocrinology and Oncology, National Research Council (IEOS-CNR), Naples, Italy; 5 Neuroimmunology Unit, IRCCS Fondazione Santa Lucia, Rome, Italy; 6 Department of Molecular Medicine and Medical Biotechnology, University Federico II, Naples, Italy

**Keywords:** Biomarkers, Diseases, Gastroenterology, Medical research, Risk factors

## Abstract

Gene expression, lipidomic and growth impairment findings suggest that the natural history of celiac disease (CD) starts before the gluten-induced immune response. Gluten intake in the first years of life is a controversial risk factor. We aimed to estimate the risk of developing CD associated with the amount of gluten intake and the serum inflammatory profile in genetically predisposed infants. From an Italian cohort of children at risk for CD, we enrolled 27 children who developed CD (cases) and 56 controls matched by sex and age. A dietary interview at 9, 12, 18, 24 and 36 months was performed. Serum cytokines (INFγ, IL1β, IL2, IL4, IL6, IL10 IL12p70, IL17, and TNFα) were analysed at 4 and 36 months. Infants who developed CD by 6 years showed an increase in serum cytokines (INFγ, IL1β, IL2, IL6, IL10, IL12p70 and TNFα) at 4 months of age before gluten introduction. CD cases ate significantly more gluten in the second year of life than controls, and gluten intake in the second year of life was strongly correlated with serum cytokines (INFγ, IL2, IL4, IL12p70, IL17) at 36 months only in CD cases. The dietary pattern of infants who developed CD was characterized by high consumption of biscuits and fruit juices and low intake of milk products, legumes, vegetables and fruits. Genetically predisposed infants who developed CD showed a unique serum cytokine profile at 4 months before gluten consumption. The amount of gluten was strongly correlated with an inflammatory profile in serum cytokines at 36 months only in infants who developed CD.

## Introduction

Celiac disease (CD) is the most common form of immune-mediated enteropathy throughout the world and is caused by an antigen-specific CD4 + T cell response to wheat gluten in genetically susceptible individuals. Although a gluten-specific Th1 response is essential for the disease, an initial innate immune response is necessary to trigger the local inflammation that drives adaptive immunity. Several triggers may unveil the disease with the appearance of autoantibodies in individuals with a strong genetic background^1^. From the Italian cohort of the longitudinal study PREVENT-CD^2^, we found several unique features in the children who developed CD by 6 years of age, before the production of anti-tissue transglutaminase antibodies (anti-tTG) or clinical symptoms. The genetic profile^3^, as well as gene expression^4^, lipidomic^5^ and clinical findings (growth^6^ and infections^7^), suggested that the natural history of CD does start before the gluten-induced immune response.

It is not yet completely clear whether the amount of gluten intake during infancy influences the subsequent risk of celiac disease: in the PREVENT-CD study, a risk associated with the amount of gluten intake could not be confirmed^8^. In contrast, three more recent studies (two in genetically at-risk children and one in a general population) found that an increased amount of gluten intake in the second year of life increased the risk of developing CD later in life^9–11^.

An intriguing hypothesis was proposed by Lindfors et al.; that is, the risk associated with gluten intake in the first years of life could be increased by a concomitant enterovirus infection^12^.

Diet could also influence systemic inflammation, and a "prudent" Mediterranean diet at one year of age is associated with a lower risk of autoimmune for celiac disease^13^. Subjects were divided into inflamed and noninflamed groups in an adult cohort according to their diet, infection history, drug use, obesity and dysbiosis^14^. Even the diet during pregnancy can influence the development of the immune system during intrauterine life, resulting in an inflamed phenotype at birth that could predispose children to chronic diseases^15^.

Gluten is well tolerated by most individuals, while in CD patients, there is a massive, proinflammatory and pathogenic immune response towards parts of the gluten proteome that is able to produce extensive T-cell-mediated damage to the small intestine. Whether this is due to a loss of tolerance or failure to establish tolerance is not known^16^. It has also been shown that gluten, and in particular α-gliadin peptide 31–43, is able to activate an innate immune response with the production of INF-alpha and IL15 in CD biopsies and fibroblasts, interfering with intracellular vesicular trafficking of a viral ligand (TLR7)^17,18^.

It is important to explore with greater accuracy the possible relationship among diet in the first years of life, the amount of gluten ingested after weaning, inflammation and the subsequent development of the disease, as this knowledge may support international recommendations and public health measures for gluten introduction in infants from celiac families carrying the set of CD-associated gene polymorphisms.

In a cohort of genetically predisposed infants, we aimed to explore the serum inflammatory profile of infants at 4 months before the introduction of gluten and at 36 months before the appearance of the disease. Then, we aimed to estimate the effect of gluten intake (from 12 to 24 months) and the dietary pattern on the risk of developing CD and its relationship with the serum cytokine profile.

## Methods

### Population and study protocol

We carried out an observational longitudinal cohort study. We enrolled 239 infants from at-risk families for CD in Italy, initially for the PREVENT-CD European project^19^ and then within the regional project NEOCEL, as previously described^7^.

The follow-up protocol for HLA DQ2- and/or DQ8-positive children included clinical and nutritional assessments and serological evaluation for CD antibodies (anti-transglutaminase and anti-gliadin) at 4, 6, 9, 12, 18 and 24 months of life and then annually until the 6th year of life. Per protocol, children avoided gluten until the age of 6 months and then introduced it gradually (500 mg at 6 months, 1000 mg at 7 months and 1500 mg at 8 months) until the age of 9 months, when they could eat gluten freely. Dietary surveys were performed by completing two forms that were included in the log-book for each scheduled visit^2^: the “one-week retrospective structured recall” and “one-day recall” forms. Gluten intake was computed by multiplying the amount of vegetable proteins contained in cereals by 0.8. Gluten was estimated as the daily amount ingested over the week before the visit, expressed as grams/day. After completing the dataset, log-books were reviewed by an independent dietitian blinded to the first evaluation to estimate the interobserver variability. Nutrients were analysed by Winfood PRO 3.24 software. Daily gluten intake was then related to body weight: grams/kg body weight/day were analysed at 9, 12, 18, 24 and 36 months of age. For quality control, the amount of gluten/kg/day estimated by the one-week survey was compared to that obtained by the one-day recall method: the difference was 0.099 (Standard Error of the Mean-SEM 0.01) at 12 months and 0.15 (SEM 0.019) at 24 months, less than 13% of the average amount estimated in the two surveys, as expected for these methods. These error estimates were not different between children with CD and controls (p > 0.3). Finally, the source of gluten and the amount and quantity of other nutrients were again scrutinized by a third investigator.

From a cohort of 239 children carrying the risk genes HLA DQ2 and/or DQ8, we diagnosed 27 cases (CD) (11.3%) at a mean age of 44.2, range 15–86 months. At the time of diagnosis, 12/27 were symptomatic (abdominal pain, diarrhoea, failure to thrive, vomiting); according to ESPGHAN criteria^20^, 25/27 cases underwent a duodenal biopsy, showing severe or subtotal mucosal atrophy in all cases, while 2/27 were symptomatic and had an anti-tTG titre 10 times above the normal limit.

Among the 212 infants with the same genetic predisposition who did not develop CD by the age of 6 years, we randomly selected 2 individuals for each CD case for a total of 56 controls (CTRLs). Controls were matched with the cases for sex, age, and familial characteristics “per protocol” study.

A total of 18/56 controls and 11/27 CD cases were previously exposed from 4 to 6 months to 100 mg of gluten during the PREVENT-CD double-blind randomized study previously described^19^.

### Ethical approval

The study was carried out according to the Helsinki II Declaration and was approved by the Ethical Committee of the School of Medicine of the University of Naples Federico II, Protocol n. 191/06. Each parent (and/or legal guardian) gave fully informed consent to the participation of their child in the study and to the use of their biological samples for research purposes.

### Genotyping

HLA typing was performed using 6 single nucleotide polymorphisms (SNPs) to identify DQ2.2, DQ2.5, DQ7 and DQ8 risk variants on the basis of strong linkage disequilibrium at HLA loci as previously described^21^. HLA-DQ risk types were predicted using the method described by Monsuur et al. and validated in several populations of European origin^22^.

### Inflammatory cytokine measurements

We analysed an available subset of cytokines from 27 CD patients and 56 CTRLs in serum samples that were collected at 4 months of age (mean 3.85, range 3–5), before gluten introduction, and after the second year of life (mean 36.2, range 24–48). Nine cytokines (INFγ, IL1β, IL2, IL4, IL6, IL10, IL12p70, IL17, and TNFα) were detected by High Sensitivity 9-Plex Human ProcartaPlex™, Luminex 200™ (Luminex Corp., Austin, TX), and XPONENT was used for data analysis. The detection limit varied for each cytokine and ranged from 0.02 ng/ml to 38 pg/ml. According to the manufacturer's specifications, the intra-assay and interassay coefficients of variation were < 10% and < 20%, respectively, for all assays. The raw values of cytokines were transformed by a decimal log because of their skewed distribution.

### Statistical analysis

Student’s t test, with Holms’ correction for multiple comparisons, and ANOVA were used to compare means. To minimize multiple testing bias, a repeated-measures analysis of variance was applied to the gluten intake over 9, 12, 18, 24 and 36 months. Logistic regression was used to estimate the ODDS associated with the increments of gluten intake over the 2^nd^ year of life. Pearson’s correlation coefficients were computed to evaluate relationships between variables. Discriminant analysis was used to estimate the capacity of serum cytokines to distinguish between CD cases and CTRLs before gluten introduction at 4 months.

## Results

### Cytokines before gluten introduction.

**Figure 1:**
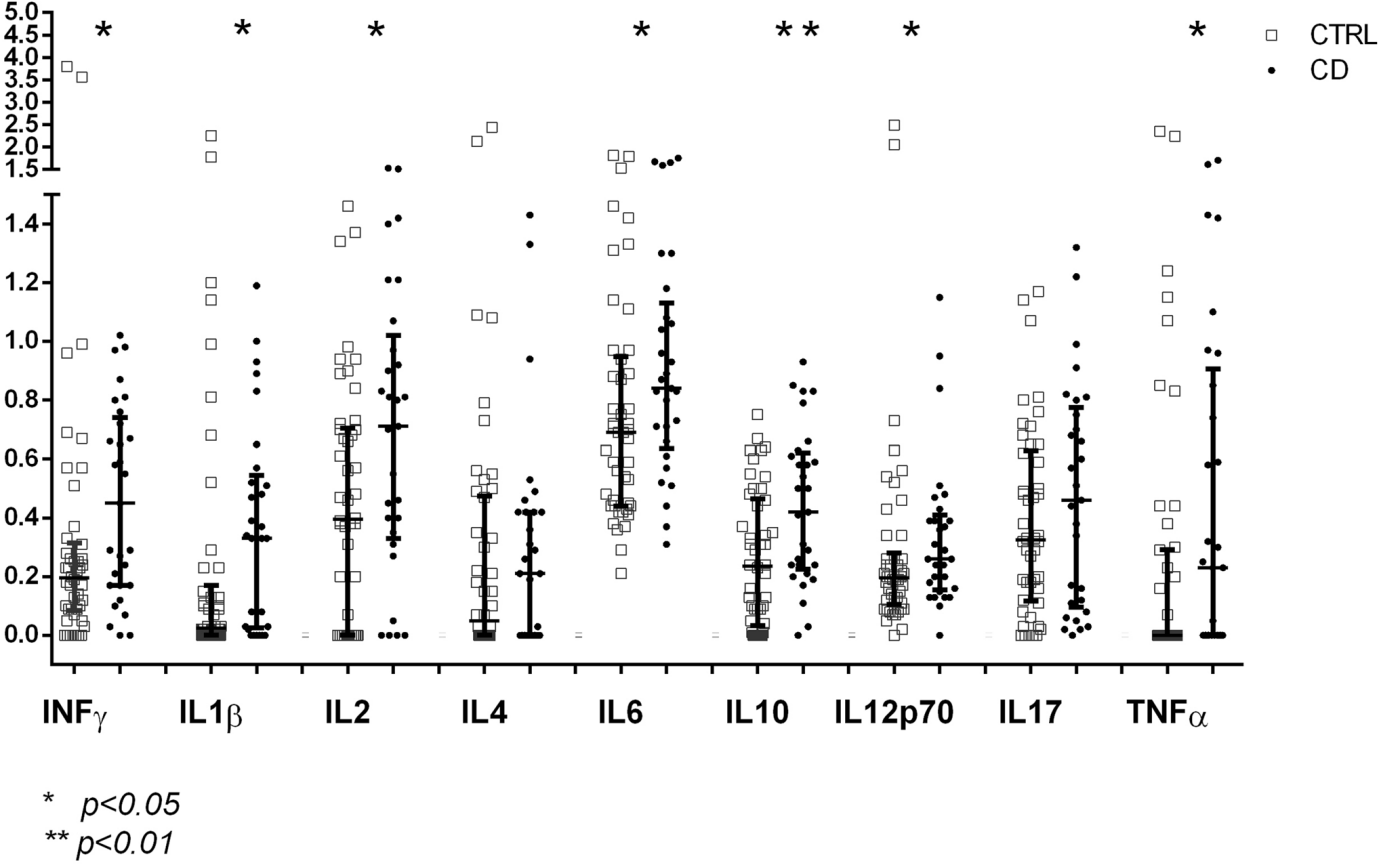
Geometric means ± 95% C.I. of cytokines at 4 months of age before gluten introduction in celiac disease (CD) cases and controls (CTRLs). *p < 0.05; **p < 0.01.

At 4 months of age, before gluten introduction, several molecules (INFγ, IL1β, IL2, IL6, IL10, IL12p70 and TNFα) showed remarkable and significant differences between the group who developed flat mucosa (CD cases) and the group who remained healthy (CTRLs). Figure 1 shows the geometric means of serum cytokines in the two groups at 4 months. All serum cytokines explored, with the exception of IL4 and IL17, showed a significant increase in their geometric means in CD cases vs. CTRLs.

Since serum cytokines are all intercorrelated, as shown by the high Pearson’s correlation coefficient in Suppl. Tab. 1 and 2, it was mandatory to run a multivariate analysis to weigh the contribution of each molecule to the differentiation between CD cases and CTRLs and deal with the multiple comparison bias. A stepwise discriminant analysis was used to estimate the capacity of each molecule to differentiate between the groups. Table 1 shows that IL10 was the best discriminating variable, followed by IL12p70, TNFα, IL4 and IL2, all contributing significantly to the differentiation between groups. INFγ, IL1β and IL6 did not contribute to the discrimination, although they were significantly different between the groups, because their variance was already explained in the model by the previously selected cytokines.

**Table 1:** Serum cytokine discriminant analysis at 4 months, before gluten introduction, between celiac disease patients (CD) and controls (CTRLs). Wilk’s lambda estimates of the capacity of each serum cytokine to discriminate between the groups once all other cytokines are considered. F is the variance ratio estimated at each step of the analysis.

STEP	Cytokines at 4 months before gluten intake	WILK’S LAMBDA	ANOVA	
			F	*p*
1	IL10	0.882	9.729	0.003
2	IL12p70	0.850	6.371	0.003
3	TNF	0.817	5.317	0.002
4	IL4	0.766	5.343	0.001
5	IL2	0.749	5.856	0.000

### Intake of gluten in the first years of life.

**Figure 2:**
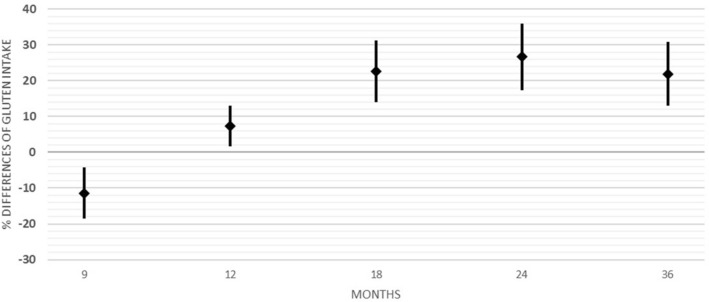
Differences between the means (± 95C.I.) in gluten intake per kg/day between celiac disease (CD) cases and controls (CTRLs) at 9, 12, 18, 24 and 36 months.

Table 2 shows the mean and confidence interval (C.I.) of the intake of gluten over the months in CTRLs and in CD cases. The size of the groups appears to differ among age groups according to the availability of complete datasets. Figure 2 shows the mean difference ± 95% C.I. between the groups as a percentage of gluten intake in CTRLs: [(Mean Gluten in CD cases –Mean Gluten in CTRLs) *100)/Mean Gluten in CTRLs]. It is clear that differences appear after the first year of life, since the study protocol (PREVENT-CD) recommended a gradual introduction of gluten in the first year of life^19^.

**Table 2:** Mean and 95% confidence interval (C. of gluten intake in grams/kg/day in controls (CTRLs) and children with celiac disease (CD). Note: in the group of CD cases, gluten at 36 months was estimated only in individuals who had not yet shown any production of anti-transglutaminase antibodies. t = T Student; df = degree of freedom.

Age (Months)	Groups	N	Mean	S.D	Low C.I	High C.I	t	df	p
9	CTRL	53	0.43	0.20	0.38	0.49	.808	72	.422
	CD	21	0.39	0.18	0.31	0.47			
12	CTRL	56	0.62	0.25	0.56	0.68	− .755	81	.452
	CD	27	0.67	0.33	0.55	0.79			
18	CTRL	54	0.63	0.26	0.56	0.70	− 2.659	79	.009
	CD	27	0.80	0.30	0.69	0.91			
24	CTRL	56	0.77	0.25	0.71	0.84	− 3.654	81	.000
	CD	27	1.04	0.42	0.88	1.20			
36	CTRL	12	0.57	0.24	1.0404	0.73	− 2.01	22	0.05
	CD	12	0.74	0.15	1.034	0.91

Since gluten intake at different ages varies little for the same individual, a repeated-measures analysis of variance (RMAV) was adopted to estimate the variance of gluten intake by month and outcome (CTRLs and CD cases), Supp. Table 3. Gluten intake increased with age in all children, but the intake in CD cases was greater than that in CTRLs at all ages. Suppl. Figure 1 shows the marginal means of gluten intake, grams/kg/day, over months of age, estimated by the RMAV model.

In the whole cohort, the intake of gluten from 12 to 24 months (over the second year of life) increased by an average of 3.56 g/day (C.I. 2.8–4.3). CD cases increased their intake by a mean of 5.31 g/day (C.I 0.3.76–6.87), while CTRLs increased their intake by 2.61 g/day (C.I. 1.88–3.35, Student’s t = -3.6 *p* = 0.001). Supp. Table 4 shows the distribution of CD cases and CTRLs according to the percentage of gluten intake in the second year of life.

We grouped the children according to the 1st and 3rd quartiles (1.71 and 5.53, respectively) of the increase in gluten intake in the second year of life and compared CD cases and CTRLs (Table 3). It is clear that 75% of CD cases showed an increase in the intake of gluten that was in the highest quartile, compared to 32% of CTRLs, producing an odds ratio of 6.37 (C.I. 1.55–26.1).

**Table 3:** CD cases and CTRLs by lowest and highest quartiles of gluten intake in the second year of life.

Group	CTRLs	CD cases
Lowest quartile	38 (68%)	7 (25%)
Highest quartile	18 (32%)	20 (75%)

ODDS RATIO = 6.37 (C.I. 1.55–26.1) χ^2^ 7.22; *p* = 0.007.

### HLA and gluten intake

In this cohort, the distribution of HLA genotypes did not differ significantly between cases and controls due to the a priori selection of children^2,7,21^. Cases and controls were grouped into 4 HLA haplotype classes, as reported previously^2,7,21^. The average amount of gluten ingested in the second year of life was not different according to the HLA risk classes (ANOVA F = 0.356, *p* = 0.23) (Suppl. Tab. 5).

### Differences in gluten intake according to relative affected

All children came from families with a first-degree relative with CD. We suspected that parents affected by CD might offer a smaller amount of gluten to their children at any age. Therefore, we compared cases and controls on the basis of the affected relative: parent or sibling.

When we compared children with an affected parent, CD cases ingested, from 12 to 24 months, an average of 0.8 g of gluten/day more than CTRLs. The difference between cases and controls in families where the affected relative was a sibling was on average (from 12 to 24 months) 0.28 g more in CD cases than in CTRLs.

In an ANOVA model, gluten intake at 18 and 24 months was significantly different between CD cases and CTRLs after correction for the relative affected.

Despite the negative results in the PREVENT-CD study about the association of early gluten exposure with the final outcome^2^, we estimated the possible confounding effect of the randomized trial with 100 mg of gluten from the 4th to the 6th month (intervention) on the final outcome in this small cohort of children. In a logistic regression model, setting sex, HLA, first-degree relative affected (parent or sibling), intervention (yes/no), and the increase in gluten intake in the second year of life (1^st^ and 3^rd^ quartile), the odds associated with the highest quartile of gluten intake (> 5.53 g/day) were 4,82 (C.I. 1.1–21, p = 0.037) compared to the lowest quartile (1.71 gr/day), while sex, parent affected, HLA and intervention did not contribute significantly to the model.

### Correlation between gluten intake at 12 months of age and serum cytokines at 36 months.

In the group of children who developed CD, at a median age of 42 months, we found a strong correlation between the amount of gluten ingested at 12 months and the cytokine level at 36 months. Specifically, it was noted that IL1β, IL2, IL4, IL12, and IL17 at 36 months were strongly correlated with the amount gluten ingested at 12 months (Suppl. Tab. 6). No significant correlation between the amount of gluten ingested at 12 months and serum cytokines at 36 months in the CTRLs was observed.

### Correlation between the total gluten intake in the second year and serum cytokines at 36 months.

**Figure 3:**
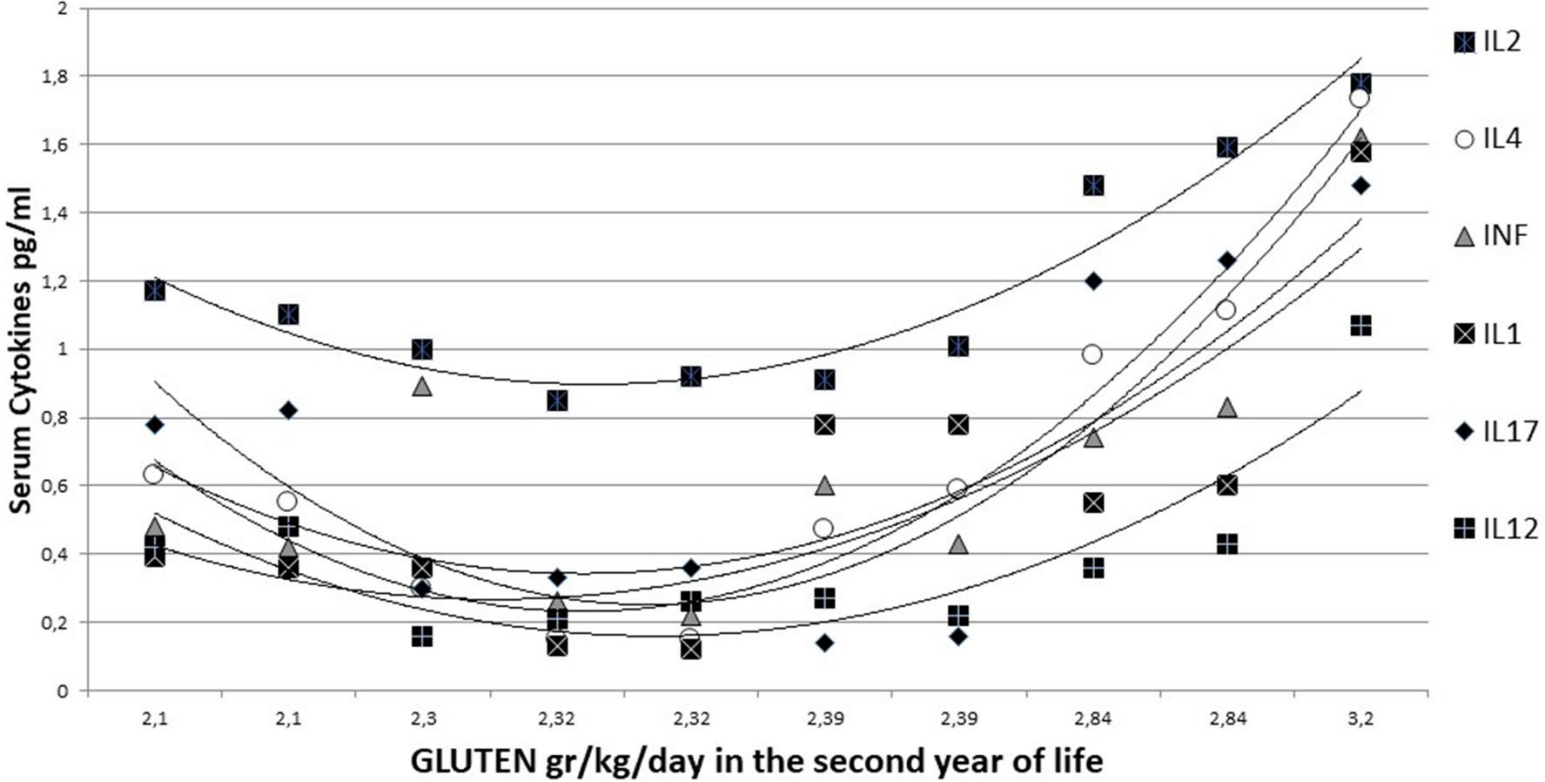
Pearson correlation coefficient ‘r’ between gluten (gr/kg/day) intake in the second year of life and serum cytokines at 36 months in the celiac disease group.

With the exception of IL6 and IL10, all serum cytokines analysed at 36 months showed a significant and strong correlation (Pearson’s coefficient r > 0.5) with gluten intake over the second year of life in children who developed celiac disease (Table 4). In controls, serum cytokines did not show any relation with gluten intake, while in CD cases, serum cytokines at 36 months showed a clear and significant strong increasing trend with the total amount of gluten eaten over the second year of life (Fig. 3). Indeed, in CD cases, serum cytokines at 36 months were correlated with gluten intake not only at 12 months but also at 18 and 24 months, as shown in Suppl. Tab. 7.

**Table 4:** Pearson’s correlation r between the total gluten ingested in the second year of life and the serum cytokine levels at 36 months in celiac disease (CD) cases and controls (CTRLs).

		*INFγ*	*IL-1β*	*IL-2*	*IL-4*	*IL-6*	*IL-10*	*IL-12p70*	*IL-17*	*TNFα*
CD CASES	r	.781*	.628	.945**	.861**	.163	.130	.746*	.930**	.379
	p	.013	.070	.000	.006	.676	.738	.021	.000	.402
CONTROLS	r	-.130	-.732**	-.578*	-.587	-.865**	-.614*	.247	-.435	.394
	p	.688	.007	.059	.058	.000	.034	.438	.158	.335

Suppl. Figure 2 shows the level of each cytokine according to the total amount of gluten eaten in the second year of life in CD cases. It is clear that the levels of cytokines show a marked increase in children with CD who ate more than 2.3 g/kg gluten in the second year of life.

### Differences in nutrient intake between 12 and 24 months

The scheduled dietary interview allowed us to estimate the amount and quality of nutrients consumed by the children over the second year. To facilitate the reading of a complex dataset, we computed the mean % differences ± 95% C.I. between the groups as [(mean of each nutrient in CD cases—mean in CTRLS) *100/mean in CTRLS]. Suppl. Figure 3 shows that, at 12 months, children who developed CD consumed larger amounts of carbohydrates (135 vs. 113 gr, t = − 2.4 *p* = 0.017), starch (43 vs. 32 t = − 1.83 *p* = 0.07) and oligosaccharides (sugars) (37 vs. 31 gr t = − 1.96 *p* = 0.06) than those who did not develop CD. CD cases consumed a very different set of fatty acids compared to CTRLs, that is, less saturated (lauric, myristic, palmitic, stearic) and monounsaturated (oleic) fats**.**

**Figure 4:**
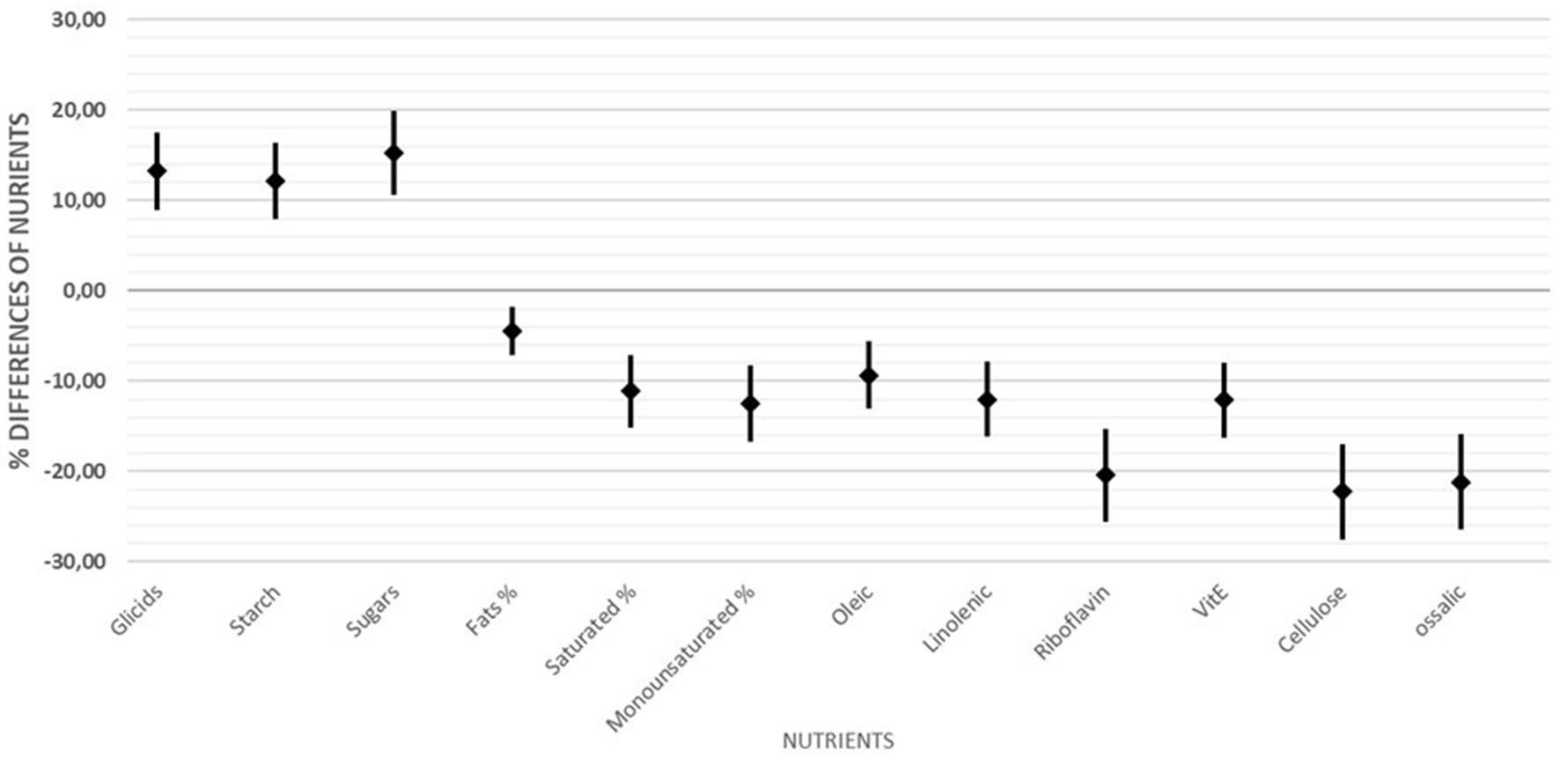
Differences in the mean intake (± 95% C.I.) of nutrients in the second year of life between celiac disease (CD) cases and controls (CTRLs).

We then analysed the total intake of nutrients over the second year of life, summing the amounts estimated at 12, 18 and 24 months (Suppl. Tab. 8). A statistically significant difference between CD cases and CTRLs (as a percentage of the amount consumed by controls) over the 2nd year of life was observed (Fig. 4).

It is clear that CD cases consumed more carbohydrates, comprising starch and oligosaccharides (sugars); less saturated and monounsaturated fats, including oleic acid; less vitamin B2 (riboflavin) and vitamin E; and less cellulose and oxalic acid than CTRLs.

When the same nutrients were analysed in relation to the weight of the child, the differences between CD cases and CTRLs were confirmed (data not shown).

We explored the personal booklet of each child to understand which food item was associated with the observed differences in the intake of nutrients (proteins, carbohydrate and lipids, etc.). We observe the following:

- The above-average intake of carbohydrates in CD cases was related to a high consumption of biscuits (up to 16/day), pizza, confectionery, cakes, snacks and fruit juices.
- The low intake of saturated and monounsaturated fatty acids in CD cases appeared to be mostly related to the low intake of milk, parmesan cheese added to soups, ricotta cheese and cheese.
- The low intake of cellulose was related to the low intake of legumes, vegetables and fruits, which is frequently observed in CD.

## Discussion

The relevance of this study is that it is the first to highlight the natural history of celiac disease, with a particular focus on what happens before the introduction of gluten and the development of the humoral response and the symptoms of this condition, to identify new therapeutic and preventive strategies for the future.

Although serum cytokines do show very large inter- and intraindividual variability^23–25^, the levels of a subset of serum cytokines at 4 months, before gluten introduction, were consistently higher in genetically predisposed infants who developed celiac disease than in matched controls. The differences were confirmed by a multivariate discriminant analysis.

This finding is in accordance with the findings from a series of our studies in genetically predisposed infants who developed CD before the age of 6 years; that is, the gene haplotypes^3^ and early gene expression^4,26^ were significantly different compared to infants who did not develop CD. Moreover, from very early in life, even before gluten introduction, the lipidomic profile has been shown to be different between the groups^5^, as is the growth pattern^6^.

These infants were gradually exposed to gluten according the PREVENT-CD protocol. At 12 months, they were free from any recommendations. The amount of ingested gluten/kg/day from 12 to 36 months was significantly greater in infants who developed CD than in those who did not up to 6 years of age. Differences appeared from the 12th month onwards and were consistent over the whole age range up to 36 months. The increase in the intake of gluten/gr/day from 12 to 24 months was approximately double in CD cases (5.31 gr/day, C.I. 3.76–6.87) compared to CTRLs (2.61 gr/day, C.I. 1.88–3.35). This finding was not influenced by affected relatives (parents or siblings) or the HLA of the infants.

The relative risk (ODDS) associated with the highest quartile of gluten intake (> 5.53 gr/day) in the second year of life was 6.37 compared to the lowest quartile (1.71 gr/day), which does suggest that the genetically predisposed infants who ate 5 or more grams of gluten/day in their second year of life had a consistently higher risk of developing celiac disease than their peers who consumed 1.7 g or less. We do not assume that these odds are directly related to the grams of gluten consumed/day, as previously reported^5^, since the 95% confidence intervals are large. This confirms that the amount of gluten ingested in the second year of life in this cohort of infants, with a homogenous genetic background, was a significant risk factor for developing celiac disease^9–11^. The time of first gluten introduction could not be studied in this PREVENT-CD cohort, where specific prescriptions were given^2^.

Regarding the diet of these children, it has been hypothesized that the increased risk of developing CD could be linked not only to gluten but also to dietary patterns being more or less adherent to the Mediterranean diet^13^. In fact, the diet of the infants who developed CD in our cohort was significantly different from those who did not: CD cases had a higher intake of carbohydrates, particularly starch and oligosaccharides (sugars), and a lower intake of legumes, vegetables and fruit than CTRLs. In other words, those who developed CD seemed to have followed in the first few years of life a diet more similar to a Western diet than a Mediterranean diet.

We have to consider that in our cohort of genetically predisposed infants, the natural history of CD began before the production of anti-tissue transglutaminase antibodies and before any clinical symptoms appeared. The children with CD started with a unique inflammatory serum cytokine profile and were exposed, just after the cessation of the per-protocol recommendations, to a significantly larger amount of gluten than their peers who did not develop the disease by 6 years.

In vitro data from our group^17,18^ suggest that the enterocytes and fibroblasts of CD subjects are constitutively inflamed (one possible cause is a delay in early-late endocytic maturation) and more prone to inflammation by gliadin peptides than those of controls.

These results suggest the presence of an “inflamed” cellular phenotype before exposure to the antigen gluten. Indeed, the genetic profile of infants who develop CD does show a series (up to 57) of differential single nucleotide polymorphisms in the gene associated with CD compared to that of infants matched for HLA who do not develop the disease up to 6 years^3^. Therefore, these children are born with a slightly different genetic background. The majority of these genes are involved in the immune response and cellular signalling, but no SNPs result in abnormal protein synthesis but are all putatively involved in gene regulation and control^27^.

A genetic predisposition associated with early gene expression, possibly modulating the serum cytokine pattern before gluten introduction, might indeed lay down an at-risk background for the massive introduction of gluten in the second year of life.

The amount of gluten ingested in the second year of life directly correlated with the serum cytokine profile at 36 months in patients with celiac disease but not in controls (Fig. 4 and Suppl. Figure 3). At 36 months, the serum profile of these infants had a clear inflammatory signature, at least one year before the appearance of anti-human transglutaminase antibodies in the serum and the development of a flat intestinal mucosa. Cytokines and chemokines are key players in the immunopathology of celiac disease; indeed, basal serum levels of some cytokines are increased in patients with active CD compared to patients on a gluten-free diet and healthy controls. Previous reports describe that the increased serum levels of some cytokines (such as IL4, IL10, IL-1α, IL-1β, IL8 and IL21) seen in CD patients are correlated with IgA anti-TG2 titres and villous atrophy^28^.

A significant and remarkable correlation coefficient does not prove a cause-effect relationship between gluten intake and inflammation, but it does allow us to reject the null hypothesis of a lack of an association between these variables. We do not wish to speculate on this finding, since developing a clear interpretation of this scenario does require exploring the profile of cytokine-producing and cytokine-expressing cells among peripheral blood lymphocytes. This work was limited by the difficulty of obtaining sufficient samples from small infants in families with a proband case who was followed up for at least 6 years.

The analysis of the dietary patterns of children who eventually developed CD suggests that not only gluten but also other ‘proinflammatory’ nutrients, such as ATI^29^, and a “Western-style diet”^30^ might be risk factors for CD. A Western-style diet in these children with CD was suggested by the intake of a high quantity of oligosaccharides (sugars) and less oleic and monounsaturated fats, as well as the scarce intake of vegetables and legumes.

We do not suggest that these experimental data, obtained in a single cohort, should be adopted to change international recommendations on infant diets, since dietary patterns are very different in different countries. However, the amount of gluten ingested in the second year of life might contribute to the unveiling of CD in genetically predisposed infants. Furthermore, there is no doubt that following weaning, a prudent diet, such as the Mediterranean diet, with possible long-term effects on the tastes and eating habits of the child^30^, with limited gluten intake in the second year of life may be a wise choice while waiting for the results proper controlled studies.

## Supplementary Information

Supplementary Information.

## Abbreviations

Anti-TG2: Anti-tissue transglutaminase antibodies
CD: Celiac disease
ATI: Amylase trypsin inhibitors
SNPs: Single nucleotide polymorphisms
