# Author Correction: Gluten consumption and inflammation affect the development of celiac disease in at-risk children

**DOI:** 10.1038/s41598-022-12636-0

**Published:** 2022-05-17

**Authors:** Renata Auricchio, Ilaria Calabrese, Martina Galatola, Donatella Cielo, Fortunata Carbone, Marianna Mancuso, Giuseppe Matarese, Riccardo Troncone, Salvatore Auricchio, Luigi Greco

**Affiliations:** 1 Department of Translational Medical Science, University Federico II, Via S. Pansini 5, 80131 Naples, Italy; 2 European Laboratory for Food Induced Diseases, University Federico II, Via S. Pansini 5, 80131 Naples, Italy; 3 Department of Clinical Medicine and Surgery, University Federico II, Naples, Italy; 4 Laboratory of Immunology, Institute for Experimental Endocrinology and Oncology, National Research Council (IEOS-CNR), Naples, Italy; 5 Neuroimmunology Unit, IRCCS Fondazione Santa Lucia, Rome, Italy; 6 Department of Molecular Medicine and Medical Biotechnology, University Federico II, Naples, Italy

Correction to: *Scientific Reports* https://doi.org/10.1038/s41598-022-09232-7, published online 30 March 2022

The original version of this Article contained an error in the spelling of the authors Renata Auricchio, Ilaria Calabrese, Martina Galatola, Donatella Cielo, Fortunata Carbone, Marianna Mancuso, Giuseppe Matarese, Riccardo Troncone, Salvatore Auricchio & Luigi Greco which were incorrectly given as Auricchio Renata, Calabrese Ilaria, Galatola Martina, Cielo Donatella, Carbone Fortunata, Mancuso Marianna, Matarese Giuseppe, Troncone Riccardo, Auricchio Salvatore & Greco Luigi. The original article has been corrected.

