# Supplementary material for: Gluten consumption and inflammation affect the development of celiac disease in at-risk children
Source: Sci Rep. 2022 Mar 30;12:5396. doi: 10.1038/s41598-022-09232-7 (PMC8968719; doi:10.1038/s41598-022-09232-7)
Supplement: Supplementary file 1 — Supplementary Information. [file 41598_2022_9232_MOESM1_ESM.docx]

**SUPPLEMENTAL TABLES’ LEGEND**

**Table S1:** Correlation among cytokines levels before gluten at 4 months in celiacs and controls. r = Spearman's rank correlation coefficient. * p<0.05 ** p<0.01

**Table S2:** Correlation among cytokines levels at 36 months before time of diagnosis in celiacs and controls. r = Spearman's rank correlation coefficient. * p<0.05 ** p<0.01

**Table S3:** Repeated Measures Analysis of Variance of gluten intake by months between celiacs and controls (CD/CTRLs).

**Table S4:** Distribution of children according to the quartile of gluten intake, and ODDs Ratio of development of the disease.

**Table S5:** Gluten intake in the second year of life by HLA status

**Table S6:** Correlation between gluten ingested at 12 months and serum concentration of cytokines at 36 months, expressed as Log10

**Table S7:** Correlation among gluten intake at 12, 24 and 36 months and cytokine levels at 36 months. r = Spearman's rank correlation coefficient. * p<0.05 ** p<0.01

**Table S8:** Means of nutrients consumed in the second year of life in celiac(CD) and controls (CTRLs)

**SUPPLEMENTAL FIGURES’ LEGEND**

**Figure S1:** Marginal means of gluten intake(gr/kg/day) over 9, 12, 18, 24 months in celiacs (CD) and controls (CTRLs) estimated by a Repeated Measures Analysis of Variance (RMAV).

**Figure S2**: Distribution of serum cytokines at 36 months (expressed as Log10) by amount of gluten intake in the second year of life in celiacs,

**Figure S3:** Differences between the means (±95% C.I.) of nutrients consumed at 12 months in celiacs and controls.

**SUPPLEMENTAL TABLES**

|  | | **IL1** | **IL2** | **IL4** | **IL6** | **IL10** | **IL12** | **IL17** | **TNF** |
| --- | --- | --- | --- | --- | --- | --- | --- | --- | --- |
| **INF** | *r* | ,966** | -0,088 | ,991** | 0,156 | ,262* | ,982** | -0,058 | ,958** |
|  | *p* | 0 | 0,454 | 0 | 0,18 | 0,023 | 0 | 0,621 | 0 |
|  | **IL1** | *r* | -0,058 | ,980** | ,238* | ,277* | ,990** | -0,029 | ,917** |
|  |  | *p* | 0,618 | 0 | 0,04 | 0,016 | 0 | 0,802 | 0 |
|  |  | **IL2** | *r* | -0,047 | ,371** | ,240* | -0,071 | ,685** | -0,005 |
|  |  |  | *p* | 0,69 | 0,001 | 0,038 | 0,543 | 0 | 0,966 |
|  |  |  | **IL4** | *r* | 0,171 | ,304** | ,990** | -0,02 | ,966** |
|  |  |  |  | *p* | 0,143 | 0,008 | 0 | 0,866 | 0 |
|  |  |  |  | **IL6** | *r* | 0,189 | 0,156 | ,521^**^ | ,232^*^ |
|  |  |  |  |  | *p* | 0,104 | 0,18 | 0 | 0,046 |
|  |  |  |  |  | **IL10** | *r* | ,278^*^ | 0,199 | ,360^**^ |
|  |  |  |  |  |  | *p* | 0,016 | 0,087 | 0,002 |
|  |  |  |  |  |  | **IL12** | *r* | -0,043 | ,926^**^ |
|  |  |  |  |  |  |  | *p* | 0,715 | 0 |
|  |  |  |  |  |  |  | **IL17** | *r* | 0,024 |
|  |  |  |  |  |  |  |  | *p* | 0,837 |

**Table S1**

|  | | **IL1** | **IL2** | **IL4** | **IL6** | **IL10** | **IL12** | **IL17** | **TNF** |
| --- | --- | --- | --- | --- | --- | --- | --- | --- | --- |
| **INF** | *r* | ,963** | ,706** | ,799** | 0,347 | 0,337 | ,840** | ,777** | ,785** |
|  | *p* | 0 | 0 | 0 | 0,105 | 0,115 | 0 | 0 | 0 |
|  | **IL1** | *r* | ,653** | ,842** | 0,326 | 0,207 | ,849** | ,704** | ,841** |
|  |  | *p* | 0,001 | 0 | 0,129 | 0,342 | 0 | 0 | 0 |
|  |  | **IL2** | *r* | ,695** | 0,255 | 0,137 | 0,413 | ,903** | ,523* |
|  |  |  | *p* | 0 | 0,241 | 0,534 | 0,05 | 0 | 0,01 |
|  |  |  | **IL4** | *r* | 0,248 | 0,154 | ,760** | ,631** | ,641** |
|  |  |  |  | *p* | 0,254 | 0,482 | 0 | 0,001 | 0,001 |
|  |  |  |  | **IL6** | *r* | ,756** | 0,034 | 0,116 | ,493* |
|  |  |  |  |  | *p* | 0 | 0,877 | 0,597 | 0,017 |
|  |  |  |  |  | **IL10** | *r* | 0,1 | 0,095 | 0,125 |
|  |  |  |  |  |  | *p* | 0,65 | 0,668 | 0,571 |
|  |  |  |  |  |  | **IL12** | *r* | ,607** | ,623** |
|  |  |  |  |  |  |  | *p* | 0,002 | 0,001 |
|  |  |  |  |  |  |  | **IL17** | *r* | ,539** |
|  |  |  |  |  |  |  |  | *p* | 0,008 |

**Table S2.**

| Source | Sum of Squares | df | MeanSquare | F | p |
| --- | --- | --- | --- | --- | --- |
| Months | 7,579 | 1 | 7,579 | 132,789 | ,000 |
| Outcome  CD/CTRLS | ,948 | 1 | ,948 | 16,609 | ,000 |
| Error | 3,653 | 64 | ,057 |  |  |

**Table S3.**

|  |  | **CTRL** | | **CD** | |  |
| --- | --- | --- | --- | --- | --- | --- |
| Percentiles of gluten intake | 10°  0,1gr/day | 10 (18,5%) | | 1 (3.7%) | |  |
|  |  |  |  |  |  |  |
|  | 25° 1,71 gr/day | 7 (13%) | | 3 (10,3%) | |  |
|  |  |  |  |  |  |  |
|  | 50° 3,02 gr/day | 12 (22,2%) | | 8 (29,6%) | |  |
|  |  |  |  |  |  |  |
|  | 75 ° 5,53 gr/day | 17 (31,5%) | | 4 (14,8%) | |  |
|  |  |  |  |  |  |  |
|  | 90° 7,65 gr/day | 8 (14,8%) | | 9 (33,3%) | |  |
|  |  |  |  |  |  |  |
| Total | | | 54 | | 27 | |
|  |  |  |  |  |  |  |

**Table S4.**

| **HLA_RISK** | **Gruppo** | **N** | **Mean Gluten intake gr/day (SD)** |
| --- | --- | --- | --- |
| DQ2/DQ2 | CTRL | 3 | 2,4 (1,53) |
|  | CD | 7 | 5,44 (2,32) |
| DQ2.5 Trans | CTRL | 11 | 3,07 (2,38) |
|  | CD | 3 | 4,13 (1,27) |
| 1/2 DQ2 | CTRL | 17 | 2,48 (2,81) |
|  | CD | 14 | 6,49 (5,28) |
| DQ2.2-DQ8 | CTRL | 22 | 2,56 (3,05) |
|  | CD | 5 | 2,54 (1,21) |

Anova: mean gluten intake by HLA risk groups by outcome.

|  | Sum of Squares | dl | Mean Square | F | p |
| --- | --- | --- | --- | --- | --- |
| Between groups | 54,477 | 3 | 18,159 | 1,514 | 0,218 |
| Intra-groups | 935,759 | 78 | 11,997 |  |  |
| Total | 990,237 | 81 |  |  |  |

**Table S5.**

|  |  | ***INF*** | ***IL1*** | ***IL2*** | ***IL4*** | ***IL6***   \|  \| \| --- \| | ***IL10*** | ***IL12*** | ***IL17*** | ***TNF*** |
| --- | --- | --- | --- | --- | --- | --- | --- | --- | --- | --- | --- |
| CONTROLS | r | -,045 | -,084 | -,235 | -,281 | -,462 | -,171 | ,381 | ,284 | -,076 |
|  | p | ,890 | ,794 | ,462 | ,402 | ,029 | ,595 | ,221 | ,371 | ,859 |
| CELIAC | r | ,353 | ,529 | ,711^*^ | ,701^*^ | ,029 | -,407 | ,575 | ,714^*^ | ,325 |
|  | p | ,286 | ,094 | ,014 | ,024 | ,933 | ,214 | ,064 | ,014 | ,394 |

**Table S6**

| **GLUTEN/Kg** | | **INF** | **IL1** | **IL2** | **IL4** | **IL6** | **IL10** | **IL12** | **IL17** | **TNF** |
| --- | --- | --- | --- | --- | --- | --- | --- | --- | --- | --- |
| **12 MONTHS** | *r* | 0,353 | 0,529 | ,711* | ,701* | 0,029 | -0,407 | 0,575 | ,714* | 0,325 |
|  | *p* | *0,286* | *0,094* | *0,014* | *0,024* | *0,933* | *0,214* | *0,064* | *0,014* | *0,394* |
| **18 MONTHS** | *r* | ,615* | 0,389 | ,687* | ,646* | 0,05 | 0,365 | 0,411 | ,625* | 0,599 |
|  | *p* | *0,044* | *0,238* | *0,02* | *0,044* | *0,883* | *0,27* | *0,21* | *0,04* | *0,088* |
| **24 MONTHS** | *r* | ,910** | ,687* | ,848** | ,766* | 0,502 | 0,464 | ,674* | ,773* | 0,237 |
|  | *p* | *0,001* | *0,041* | *0,004* | *0,027* | *0,168* | *0,209* | *0,047* | *0,014* | *0,61* |

**Table S7**

|  | **group** | **N** | **Mean** | **Std. Dev.** | **St. Error of the Mean** |
| --- | --- | --- | --- | --- | --- |
| **Carboidrates%Cal** | CTRL | 156 | 44,570 | 6,9947 | ,5496 |
|  | CELIAC | 27 | 47,049 | 6,6007 | ,7779 |
| **Glicids** | CTRL | 156 | 123,193 | 34,7215 | 2,7280 |
|  | CELIAC | 27 | 139,471 | 49,4954 | 5,8331 |
| **Starch** | CTRL | 157 | 43,888 | 25,8173 | 2,0604 |
|  | CELIAC | 27 | 49,230 | 31,7101 | 3,8174 |
| **Sugars** | CTRL | 156 | 34,056 | 18,6849 | 1,4680 |
|  | CELIAC | 27 | 39,242 | 19,9289 | 2,3651 |
| **Fats % Cal** | CTRL | 156 | 37,661 | 6,5801 | ,5170 |
|  | CELIAC | 27 | 35,979 | 6,0119 | ,7085 |
| **SAFA % Cal** | CTRL | 156 | 11,752 | 5,0054 | ,3933 |
|  | CELIAC | 27 | 10,449 | 4,4614 | ,5258 |
| **MUFA % Cal** | CTRL | 156 | 12,906 | 5,0141 | ,3939 |
|  | CELIAC | 27 | 11,303 | 5,1116 | ,6024 |
| **Oleic** | CTRL | 156 | ,9956 | ,55237 | ,04422 |
|  | CELIAC | 27 | ,9028 | ,56552 | ,06759 |
| **C18:3 linolenic** | CTRL | 159 | ,323 | ,1530 | ,0121 |
|  | CELIAC | 27 | ,284 | ,1323 | ,0160 |
| **Riboflavin** | CTRL | 156 | 1,206 | ,8237 | ,0647 |
|  | CELIAC | 27 | ,960 | ,4089 | ,0482 |
| **VitE** | CTRL | 156 | 4,719 | 2,3898 | ,1883 |
|  | CELIAC | 27 | 4,149 | 2,3129 | ,2726 |
| **Cellulose** | CTRL | 156 | ,796 | ,5846 | ,0570 |
|  | CELIAC | 27 | ,619 | ,2830 | ,0437 |
| **Ossalic** | CTRL | 156 | 57,073 | 111,6268 | 11,6379 |
|  | CELIAC | 27 | 20,621 | 16,3595 | 2,4948 |

**Table S8**

**SUPPLEMENTAL FIGURES**


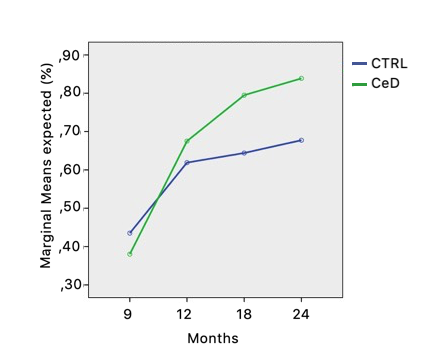


**Figure S1**.

**INF IL1**

**
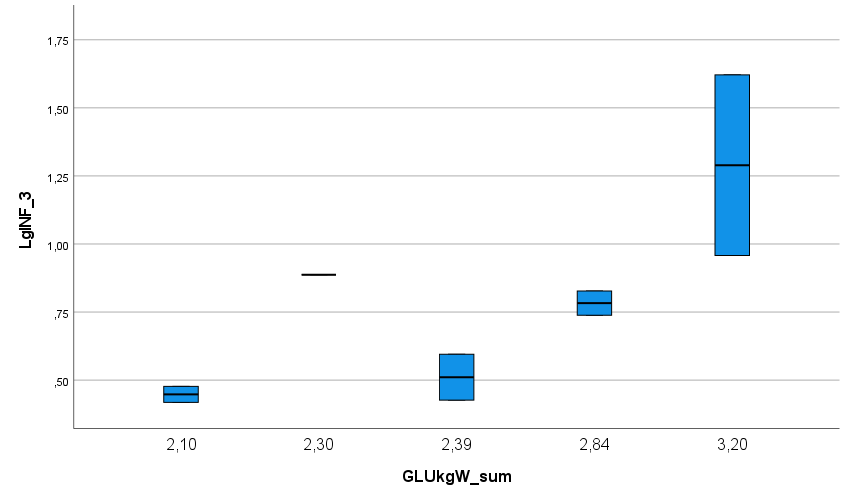

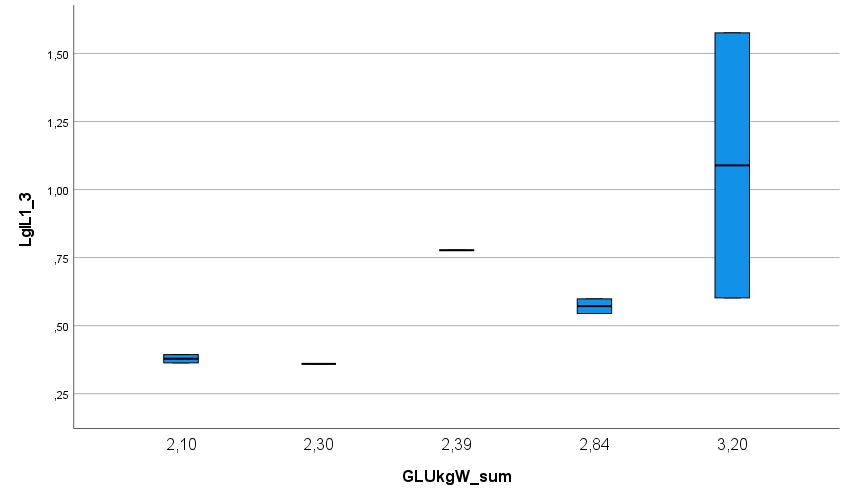
**

**IL2 IL4**

**
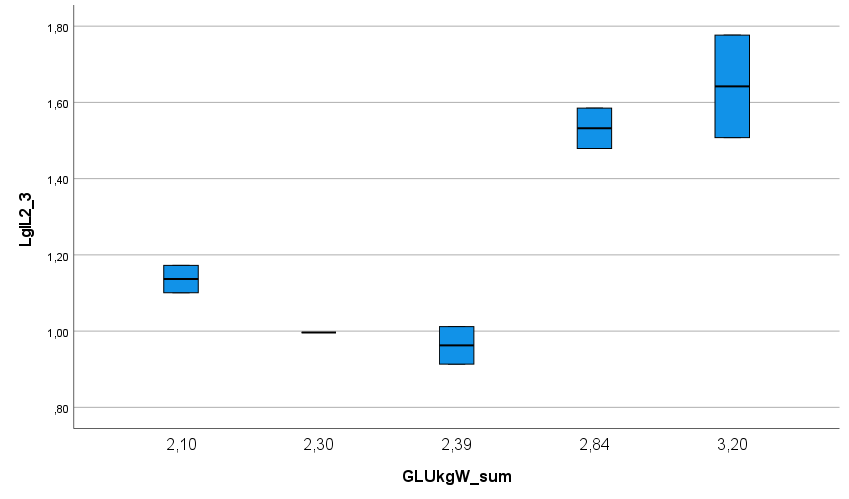

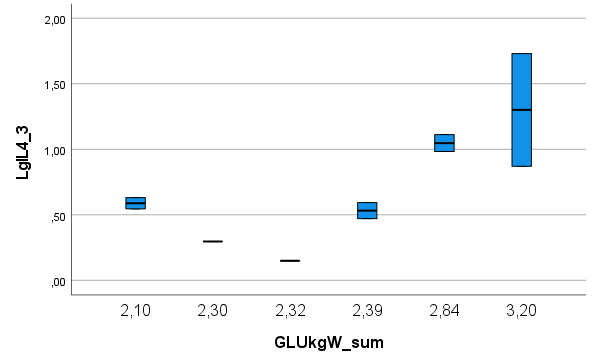
**

**IL12 TNF**

**
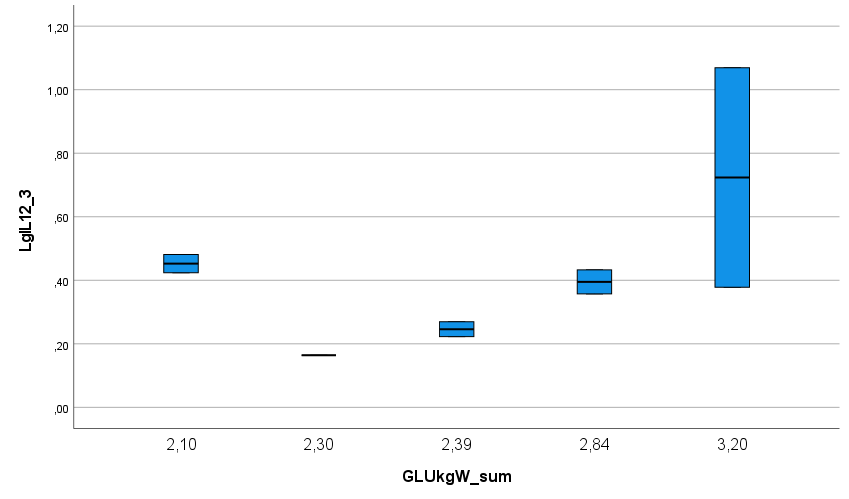

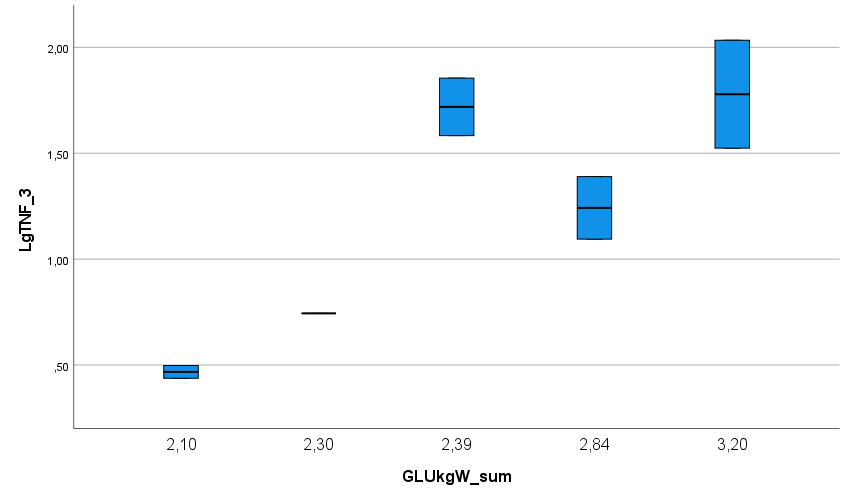
**

**Figure S2**

**
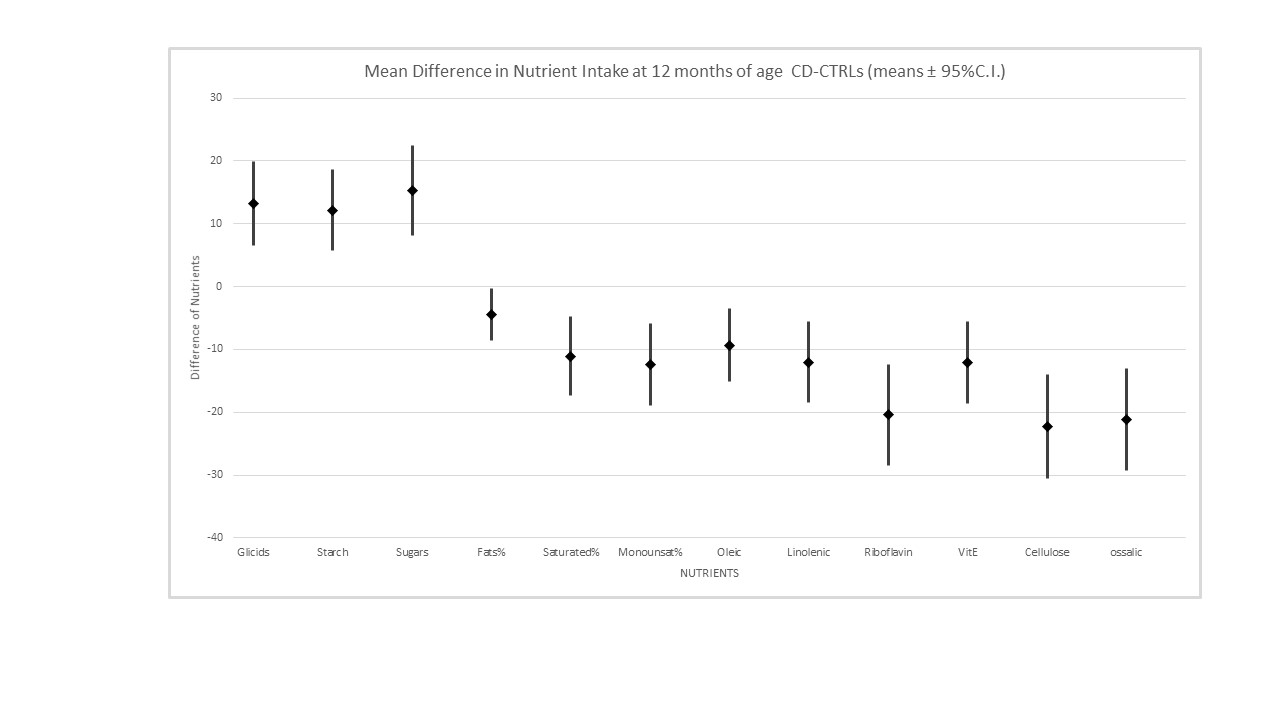
**

**Figure S3.**
